# Metabolic costs of physiological heat stress responses - Q_10 _coefficients relating oxygen consumption to body temperature

**DOI:** 10.1186/2046-7648-4-S1-A103

**Published:** 2015-09-14

**Authors:** Bernhard Kampmann, Peter Bröde

**Affiliations:** 1 Department of Safety Engineering, Bergische Universität Wuppertal, Germany; 2 Leibniz Research Centre for Working Environment and Human Factors (IfADo), Dortmund, Germany

## Introduction

Q_10 _describes the influence of temperature on physiological processes as the ratio of the rate of a physiological process at a particular temperature to the rate at a temperature 10 °C lower [1]. In terms of rates of oxygen consumption (VO_2_) related to rectal temperatures (t_re_), this can be written as [2]:

(1a) $${\text{Q}}_{10}={\left(\text{V}{\text{O}}_{2}/\text{V}{{\text{O}}_{2}}_{,\text{ref}}\right)}^{10/\left(\text{tre}-\text{tre,ref}\right)}$$

or equivalently,

(1b) $$\text{V}{\text{O}}_{2}=\text{V}{\text{O}}_{2,\text{ref}}\;.\;{Q_{10}}^{\left(\text{tre}-\text{tre,ref}\right)/10}$$

Q_10 _varies between 2 and 3 in biological systems [2], and Q_10 _= 2 is applied in modelling the rate of metabolic heat production in relation to body temperature [3,4]. This paper aims to determine Q_10 _for the influence of body temperature on oxygen consumption for light work in warm environments.

## Methods

Data originated from 216 laboratory experiments [5] consisting of individual series of 14 to 39 trials performed by eleven acclimatised semi-nude young males (I_cl_=.1 clo) who walked 4 km.h^-1 ^on the level for at least 3 hours under different combinations of water vapour pressure (range 0.3 - 5.2 kPa) and air temperature (range 20 - 55 °C) with air velocity of 0.3 m.s^-1 ^and mean radiant temperature equal to air temperature. Mean values of t_re _and VO_2 _over the third hour of exposure were submitted to linear regression analyses, which were performed separately for the 11 individual series relating VO_2 _directly to t_re _and also using the logarithmised Eq. 1b (with t_re,ref _= 36.8 °C). Overall regression parameters were calculated by random coefficient linear mixed models considering the correlation within the individual series. Q_10 _coefficients were obtained as the exponentiated slopes of the fitted logarithmised Eq. 1b.

## Results

**Figure 1:**
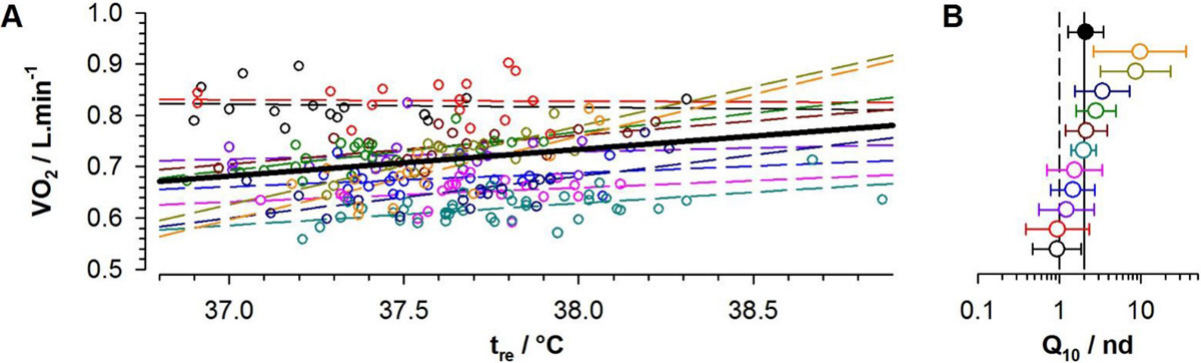
VO_2 _related to t_re _with overall regression (solid, VO_2 _= 0.671+0.052(t_re_-36.8)) and individual lines (dashed) for 11 participants (A), and Q_10 _with 95% CI for 11 individuals (open symbols) and for the total sample (filled symbol) with reference lines indicating the neutral value (Q_10 _= 1, dashed) and Q_10 _= 2 (solid) (B).

Regression analyses showed a statistically significant (p < 0.01) increase of VO_2 _with t_re _(Figure 1A) with inter-individually varying slopes, which resulted in Q_10 _values varying largely between 1 (indicating no influence of t_re _on VO_2_) and 10 (Figure 1B). The overall Q_10 _was 2.1 with 95% confidence interval (CI) 1.3 - 3.5.

## Discussion and conclusion

The results support the setting Q_10 _= 2 [3,4] under steady state conditions for light work in the heat, however, considerable intra- and inter-individual variability was observed.

Thus, the data base should be extended, also towards other workloads and populations (female, elderly).
